# Supplementary material for: Aortopathies in mouse models of Pompe, Fabry and Mucopolysaccharidosis IIIB lysosomal storage diseases
Source: PLoS One. 2020 May 19;15(5):e0233050. doi: 10.1371/journal.pone.0233050 (PMC7236983; doi:10.1371/journal.pone.0233050)
Supplement: Attachment — Submitted filename: Response to Reviewers.docx [file pone.0233050.s002.docx]

Corresponding Author:

Giuseppe Limongelli, MD, PhD, FESC

Inherited and Rare Cardiovascular Disease Clinic

Department of Translational Medical Sciences

University of Campania “Luigi Vanvitelli”

Via L. Bianchi 1 c/o Monaldi Hospital, AORN Colli

Tel: 0817062815

Email: [limongelligiuseppe@libero.it](mailto:limongelligiuseppe@libero.it)

26^th^ of February 2020

Dear Editor,

Thank you for the opportunity to revise our paper. We believe that, following reviewer’s suggestions, the paper has much improved. Moreover, we ask you to include 2 new coauthors (Valeria De Pasquale, Salvatore Esposito), since the additional pathological and immunohistochemical data were performed in their labs.

We hope you will consider it for publication in PLOS One.

Kind regards,

Giuseppe Limongelli

**Response to reviewers**

**Reviewer #1**

**Question n. 1 - Major**

This is an interesting manuscript that highlights an aspect of LSDs that is not often considered. I wonder whether the authors can provide a little more data on parameters that are relevant for the aortopathies that they observe? For example cell biological analysis of storage material/lysosomal pathology? Other minor comments are listed below.

*Answer n. 1 – Major*

*Thank you for your comment. As suggested, in the revised version of the manuscript we have provided available data on the histochemical analysis of the KO mice (NAGLU-/- and GAA-/-), describing unique characteristics of the semilunar valve and vessel in these aortic models. We think that this evidence significantly strengthens the paper.*

*We specified in the text:”* *H&E staining and PAS sections in GAA-/- mice evidenced an aortic wall with mild disorganization of lamellar units for the presence of many vacuoles containing fine granular or amorphous material in their reduced inter-lamellar space. The aortic valve was not thickened, nor accumulation was found (data not showed).*

*H&E staining and PAS sections in in NAGLU-/- mice confirmed the aortic valve defects detected by echocardiography at the time point examined. NAGLU-/- mice exhibited significant aortic cuspid thickening with excess/redundant tissue present in the valves as demonstrated by Alcian blue-PAS staining. By using the H&E staining, NAGLU-/- aortic valve cuspids exhibited a disruption of the normal collagen/proteoglycan boundary interfaces observed in WT animals”*

**Question n. 2 - Minor**

- Literature citations and discussion are incomplete, no mentioning has been made on aortic stiffness in human patients. These are relevant to this manuscript.

*Answer n. 2 - Minor*

*Thank you for your comment. As you suggested, we expanded the literature citations and discussion.*

**Question n. 3 - Minor**

- The introduction is too short in my opinion. The question: 'aortopathies…have not been investigated in pre-clinical models…' is not well explained. Why is this study relevant? It is in my opinion, but please make this more clear and provide a more comprehensive reasoning to introduce the topic

*Answer n. 3 - Minor*

*Thank you for your comment. As suggested, we expanded the introduction, focusing on cardiac and aortic involvement in patients with LSDs, to clarify the aim and the importance of the present study.*

**Question n. 4 - Minor**

- please provide information on the genetic background of the mice and corresponding wild type mice.

*Answer n. 4 - Minor*

*Thank you for your comment. As suggested, we have provided the genetic background of the mice models studied. We specified in the materials and methods as follows: “MPS IIIB knockout mice (NAGLU-/-) were generated by insertion of neomycin resistance gene into exon 6 of NALGU gene on the C57/BL6 background .GAA-/- murine model was created by insertion of plasmid containing both the neomycin-resistance gene and the herpes virus thymidine kinase gene in the pBluescript vector. Six independent cell lines containing the disrupted GAA allele were used to make chimeras that were bred to C57BL/6 females to generate heterozygous mice (F1). Fabry KO mice were generated by a targeted disruption of the α-GAL A gene on the C57Bl/6N back-ground. α-GAL A and wildtype (WT) mice were genotyped by PCR.”*

**Question n. 5 - Minor**

- Please provide the data for the weight of the ko mouse models at analysis

*Answer n. 5 - Minor*

*Thank you for your comment. We have provided the weight of the mouse models investigated. In particular, we specified in the text:”* *The weight of KO mouse models was not significantly different compared to WT (GLA-/- 29.2 ±2.2, NAGLU-/- 28.9 ±0.7, GAA-/- 28 ±2.0, WT 29 ±1.0).”*

**Question n. 6 - Minor**

- The figures are redundant with the tables and could be removed

*Answer n. 6 - Minor*

*Thank you for your comment. As suggested, we have removed the figures.*

**Question n. 7 - Minor**

- how was multiple testing corrected for?

*Answer n. 7 – Minor*

*Thank you for your comment. To compare the aortic parameters in different mouse models we used t-test or ANOVA (when appropriate). Since the groups were similar, multivariate analysis was not performed.*

**Reviewer #2**

**Question n. 1 - Major**

The paper by Belfiore et al describes the measurement of some aortic parameters by using echocardiography in 3 different mouse models of LSD, in particular Pompe Disease, Fabry Disease and Sanfilippo IIIB. Although such evaluation, conducted on each single model and also among the 3 models, showed some statistically significant differences between each model vs. wt mice as well as among the models, and although the Authors state this is the first study on this specific topic in these LSD models, this reviewer finds the study not particularly interesting due to the already available data on the patients affected by the same diseases, who can be much more informative than the mouse models. In my opinion, mouse models are worthy to be tested and used when similar data are nor recoverable from the clinics, also taking into consideration the important differences that might sometimes be registered between the models and the human subjects. In addition, the very limited number of mice examined further reduces the interest of the paper.

In my opinion, the paper is not worthy of publication.

Maybe Authors should have complemented the presented data by pathologically characterizing the cardiac region examined and by providing data obtained from the same mice following treatment, as Authors themselves state in the conclusion of the manuscript.

*Answer n. 1 - Major*

*Thank you for your comment. We think that the paper provides important information in the field of LSDs, since the knowledge about the aortic involvement in these conditions are scant. As suggested, we have provided the histochemical analysis of the available KO mice (NAGLU-/- and GAA-/-) describing unique characteristics of the semilunar valve and vessel in these aortic models. We think that this evidence significantly strengthens the paper.*

*Unfortunately, we were not able to investigate the effects of therapy on our models. This aim, although really interesting, would take years and efforts to be completed.*

*We specified in the text:”* *H&E staining and PAS sections in GAA-/- mice evidenced an aortic wall with mild disorganization of lamellar units for the presence of many vacuoles containing fine granular or amorphous material in their reduced inter-lamellar space. The aortic valve was not thickened, nor accumulation was found (data not showed).*

*H&E staining and PAS sections in in NAGLU-/- mice confirmed the aortic valve defects detected by echocardiography at the time point examined. NAGLU-/- mice exhibited significant aortic cuspid thickening with excess/redundant tissue present in the valves as demonstrated by Alcian blue-PAS staining. By using the H&E staining, NAGLU-/- aortic valve cuspids exhibited a disruption of the normal collagen/proteoglycan boundary interfaces observed in WT animals”*
