# Supplementary material for: Aortopathies in mouse models of Pompe, Fabry and Mucopolysaccharidosis IIIB lysosomal storage diseases
Source: PLoS One. 2020 May 19;15(5):e0233050. doi: 10.1371/journal.pone.0233050 (PMC7236983; doi:10.1371/journal.pone.0233050)
Supplement: Attachment — Submitted filename: Response to reviewers (10.04 def).docx [file pone.0233050.s003.docx]

**Corresponding Author:**

Giuseppe Limongelli, MD, PhD, FESC

Inherited and Rare Cardiovascular Disease Clinic

Department of Translational Medical Sciences

University of Campania “Luigi Vanvitelli”

Via L. Bianchi 1 c/o Monaldi Hospital, AORN Colli

Tel: 0817062815

Email: [limongelligiuseppe@libero.it](mailto:limongelligiuseppe@libero.it)

11^th^ of April 2020

Dear Editor,

Thank you for the opportunity to revise our paper. We believe that, following reviewer’s suggestions, the paper has much improved.

We hope you will consider it for publication in PLOS One.

Kind regards,

Giuseppe Limongelli

**Response to Reviewers.**

**Reviewer #1:**

The authors have significantly improved the manuscript. The histochemical data are partially informative. In the legend to Fig1, no A or B has been described. The quality of Figures 2 (out of focus) and 3 (no details, unclear phenotype, very small and damaged preparations) is not so good. Do the authors have better pictures? Otherwise I would consider removing these. The English needs to be revised.

*Thank you for your comment. Pictures have been modified and improved in their quality. English have been revised all over the text.*

**Reviewer #2:**

1) Although the manuscript was slightly revised, the Authors did not explain why they evaluated a so small number of animals. In addition, Authors did not specify in each Table or Figure the number of animals analyzed in each analysis and did not indicate which Wild Type mice were analyzed.

*Thank you for your comment. This study was essentially designed to describe the aortic phenotype in different mouse models of lysosomal storage disease. To this aim, we believe that sample size was efficacious to verify our hypothesis. Moreover, according to your suggestion, the number of animals analyzed in each analysis was specified in each Table or Figure.*

It is known that WT mice deriving from different colonies may not share similar features. Thus, each model should include for comparison wild type mice deriving from the same colony, whereas from the manuscript it seems that Authors used a general group of wild type mice with no specific reference to each colony.

*Wild type mice were absolutely derived from the same colony of their correspondent LSD mouse model. We corrected the text all over indicating that phenotypic comparisons have been performed against wild type mice deriving from the same colony of their correspondent KO mice.*

2) All along the manuscript MPS IIIB is called MPS, which is a misleading definition, since features of MPS IIIB may be not extendable to all MPS. We know for example that as for other cardiologic features, as valvulopathies or others, not all MPSs present the same degree of involvement or even the same presence/absence in the patients along their pathological progression, so to ascertain that the described features, related to aortopathies, were only identified in the MPS IIIB mouse model is mandatory. This should be described also in the discussion in more details.

*In agreement with reviewer suggestion, we modified all along the manuscript the meaning of MPS by specifying that our model is referred to the specific MPS IIIB disease. We specified this also in the discussion section.*

3) Why did the Authors analyze animals at 12 months of age? Authors should explain why they decided to evaluate all animals at the same age while different models may show pathological signs/symptoms at different ages. Very likely at 12 months of age all models present a very pathological phenotype. Which was the disease progression in the different models?

*Thank you for your important comment. Your assumption is right, since at 12 months of age all models are very likely to show pathological phenotype (including a progressive phenotype as aortopathy). The main aim of this study was to describe the aortic phenotype in different mouse models of lysosomal storage disease. On the other hand, this pilot study was not designed to describe aortic progression in different models and to test different therapies (i.e. ERT, chaperone, genetic therapy, etc). Therefore, we do not have data on different aortic parameters progression, and we stated this in the study limitation.*

**Reviewer #3:**

In this work, the Belfiore et al have investigated the presence and characteristics of aortopathy related to three lysosomal storage disorder models Pompe Disease (PD), Fabry disease (FD) and mucopolysaccharidoses (MPS). In a series of experiments the authors have reported differences and in the revised version the authors have supplied some additional experimental evidence to answer one of the reviewers.

The second reviewer who recommended rejection based this decision on the small number of animals used in the experiments and not providing data following ERT treatment.

Although it is understandable that providing data following treatment might take too long it is not clear to this reviewer whether all the different mouse strains were tested with wild-types from each respective background. If this has not been done then it would be important to include it in a eventually revised version of the manuscript.

*Thank you for your comment. Wild type mice were absolutely derived from the same colony of their correspondent LSD mouse model. We corrected the text all over indicating that phenotypic comparisons have been performed against wild type mice deriving from the same colony of their correspondent KO mice.*
